# Human pluripotent stem cell derived HLC transcriptome data enables molecular dissection of hepatogenesis

**DOI:** 10.1038/sdata.2018.35

**Published:** 2018-03-13

**Authors:** Wasco Wruck, James Adjaye

**Affiliations:** 1 Medical Faculty, Institute for Stem Cell Research and Regenerative Medicine, Heinrich Heine University, 40225 Düsseldorf, Germany

**Keywords:** Stem-cell research, Stem-cell differentiation, Transcriptomics, Microarray analysis

## Abstract

Induced pluripotent stem cells (iPSCs) and human embryonic stem cells (hESCs) differentiated into hepatocyte-like cells (HLCs) provide a defined and renewable source of cells for drug screening, toxicology and regenerative medicine. We previously reprogrammed human fetal foreskin fibroblast cells (HFF1) into iPSCs employing an episomal plasmid-based integration-free approach, this iPSC-line and the hESC lines H1 and H9 were used to model hepatogenesis *in vitro.* Biochemical characterisation confirmed glycogen storage, ICG uptake and release, urea and bile acid production, as well as CYP3A4 activity. Microarray-based transcriptome analyses was carried out using RNA isolated from the undifferentiated pluripotent stem cells and subsequent differentiation stages- definitive endoderm (DE) hepatic endoderm (HE) and HLCs. K-means identified 100 distinct clusters, for example, *POU5F1*/*OCT4* marking the undifferentiated stage, *SOX17* the DE stage, *HNF4α* the HE stage, and *ALB* specific to HLCs, fetal liver and primary human hepatocytes (PHH). This data descriptor describes these datasets which should be useful for gaining new insights into the molecular basis of hepatogenesis and associated gene regulatory networks.

## Background & Summary

The implementation of a well-characterized renewable source of hepatocytes differentiated from iPSCs and hESCs provides a powerful *in vitro* model system for analysing the molecular mechanisms associated with hepatogenesis. Several essential initiators of hepatogenesis such as fibroblast growth factor 2 and 4 (FGF4 and FGF2)^1,2^, bone morphogenic protein (BMP2)^3^, hepatocyte growth factor (HGF), oncostatin M and dexamethasone^4^ have already been described. These factors are sequentially supplemented into the media during the course of the differentiation process.

Besides detoxification the liver is responsible for a number of essential functions e.g. the uptake and storage of glycogen, various metabolic functions, synthesis of bile acids and production of plasma proteins. Available liver cellular models have disadvantages: (i) liver biopsy derived primary human hepatocytes (PHH) cannot be expanded for long periods *in vitro*, are often obtained from diseased individuals and are difficult to obtain in sufficient quantities^5,6^, (ii) transformed, permanent cell lines, such as HepG2 and HepaRG, have cancer phenotypes which are significantly diverged from normal primary hepatocytes^7–9^. A potential alternative could be the differentiation into hepatocyte-like cells. Although hepatocyte-like cells (HLCs) derived from iPSCs are not fully mature compared to liver biopsy derived adult hepatocytes they are endowed with many advantages, for example easily generated from iPSCs, known genetic background and disease states thus optimal for disease modelling *in vitro,* toxicology studies and drug screening. iPSC-based cellular models have already been employed in several studies for drug screening, toxicology studies and disease modeling^10–14^.

The liver develops in a stepwise process *in vivo*: first, competence is established in the foregut endoderm in response to signals emanating from cardiac mesoderm, thereafter liver-specific gene expression is initiated^15^. The differentiation of hiPSCs and hESCs also proceed via the intermediate step of definitive endoderm, the bipotential hepatic endoderm, then maturation into HLCs^16^. Distinct stage specific changes in the associated transcriptional regulatory networks control the different phases of hepatogenesis^17^. Wang *et al.* describe a developmental progression from unmarked chromatin to poised chromatin and then to histone H3K27 acetylation which is accompanied by specific transcription factor classes^18^. They suggest FOXA transcription factors - known as pioneer factors facilitating the unwinding of chromatin - to play a role at poised enhancers while lineage-specific factors such as PDX1 for pancreatic and HNF4α for hepatic lineage drive the poised to an active enhancer state^18^.

Attaining maturation comparable to primary hepatocytes is still one of the most challenging issues associated HLC differentiation. Knowledge on HNF4α, as major transcription factor regulating hepatic differentiation and maturation has already been described^15^. Additionally Li *et al.* reported that HNF4α lies upstream of the transcription factors HNF1α and PXR suggesting it might initiate a cascade of gene regulatory networks driving hepatocyte differentiation^15^. In our publication related to the hESCs and hiPSC dataset pertinent to this data descriptor we confirmed expression of maturation markers such as *ALB, HNF4α, HNF1α* and *TTR*^16,17^.

The data described consists of microarray gene expression data from hESCs and hiPSCs differentiated into HLCs via the DE and HE stages and also fetal liver and primary human hepatocyte samples as reference. Although transcription factors central to hepatogenesis have been described, the datasets described here will enable a more detailed analyses of gene regulatory networks associated with modelling hepatogenesis using pluripotent stem cells.

## Methods

### Human ES and iPS cells culture

Human ES cell lines H1 and H9 (WiCell Research Institute, Madison, Wisconsin) from passage 39 to 66 were maintained under sterile conditions in a humidified incubator in a 5% CO2-95% air atmosphere at 37 °C (INNOVA CO-170 Incubator, New Brunswick Scientific). In a routine culture cells were maintained on Matrigel^®^ in conditioned media (CM)^19^. Under these culture conditions, hESCs were confirmed to stain positive for OCT4, SSEA-4, TRA-1-60, and TRA-1-81 (ES Cell Characterization Kit, Chemicon). Before initiating the differentiation cells were washed with PBS without Ca2+Mg2+ (Gibco, Invitrogen).

Cell culture of iPS cells which were derived from Human neonatal foreskin fibroblasts HFF1 is described in Matz *et al.*^17^.

### Differentiation into hepatocyte-like cells (HLCs)

The derivation of HLCs from the hESC lines H1 and H9^16^ followed protocols described by Hay *et al.*^20^ and Agarwal *et al.*^21^. RNA samples were extracted after each step of the differentiation protocol.

Differentiation of iPS cells into HLCs^17^ followed in large parts the protocol described by Jozefczuk *et al.*^16^.

**Figure 1:**
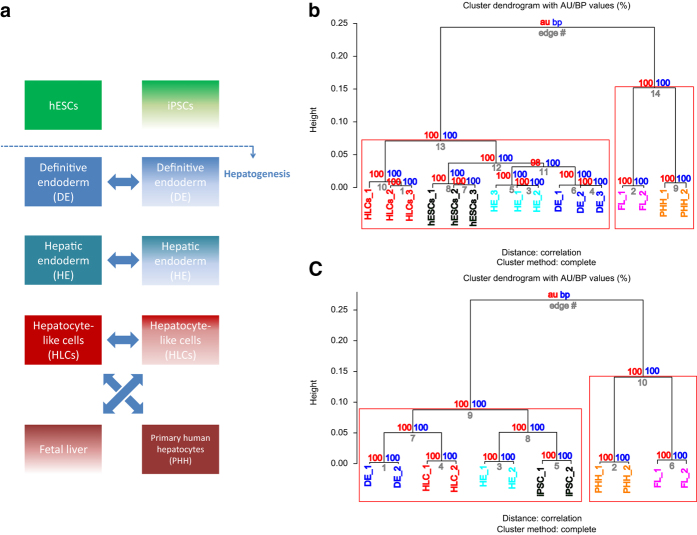
Comparison of hepatic differentiation of iPSCs and hESCs. (**a**) Scheme of comparative hepatic differentiation of iPSCs and hESCs. iPSCs and hESCs are differentiated into HLCs which can be compared versus each other and versus fetal liver (FL) and PHHs. Also the intermediate stages DE and HE are captured and thus can be subjected to comparative analysis. Hierarchical clustering of hESC (**b**) and iPSC (**c**) differentiation into HLCs was performed via pvclust using 1000 bootstrap samples. „au“ (approximately unbiased) is computed by multiscale bootstrap resampling and „bp“ (bootstrap probability) by normal bootstrapping. Red rectangles mark clusters with AU larger than 95%. Thus the dendrogram is with one exception in hESC (98%) at 100% supported by data. All replicates cluster together. HLCs cluster apart from hESCs/iPSCs, DE and HE. Fetal liver and PHH cluster together and separated from the hESC/iPSC-derived hepatic differentiation stages.

The overall experimental design of this study is illustrated in Figure 1a. Two pluripotent stem cells lines (hESC- H1 and H9) and fetal foreskin derived iPSC were used. Both proceeded via the intermediate DE and HE stages to HLCs and then compared to commercially bought RNA from fetal liver (Stratagene, MVP Total RNA: tissue from single male donor, 18th week of gestation; positive control for the iPSC-based differentiations: Clontech, #636540) and adult liver biopsy-derived primary human hepatocytes-PHH (Ready Heps Fresh Hepatocytes; Lonza, 65-year old male of Asian origin; positive control for the iPSC-based differentiations: Clontech, #636531).

### Illumina BeadChip hybridisation

Biotin-labelled cRNA was produced by means of a linear amplification kit (Ambion, Austin, TX, USA) using 500 ng of quality-checked total RNA as input. Chip hybridisations, washing, Cy3-streptavidin staining, and scanning were performed on an Illumina BeadStation 500 platform (Il-lumina, San Diego, CA, USA) using reagents and following protocols supplied by the manufacturer. cRNA samples were hybridised in biological triplicates on HumanRef-8 Expression BeadChips. The following samples were hybridized: Undifferentiated cells (H1 cell line), DE (definitive endoderm)-differentiated cells, HE (hepatic endoderm)-differentiated cells and hepatocyte-like cells (HLCs) derived with two independent protocols^20,21^.

Differentiation experiments of iPSCs were hybridised on Illumina HumanHT-12 BeadChips. For details see the Methods description in Matz *et al.*^17^.

The summary of bead-level data to bead-summary data was carried out using the manufacturer's software BeadStudio 3.0 (Illumina) for hESC and iPSC differentiation experiments. Table 1 provides an overview of all samples used for this study.

### Data analysis and statistical methods

For further analysis, the bead-summary data saved in the BeadStudio was imported into the Bioconductor environment^22^ and quantile normalized using the bioconductor package lumi^23^. Global gene expression similarities within biological replicates and between dedicated differentiation stages, pairwise Pearson correlation coefficients were calculated for all samples. Cluster analyses were performed using the R/Bioconductor environment^22^ and the package pvclust^24^ using n=1000 for bootstrap sampling. k-means clustering was employed to identify clusters of genes with similar gene expression changes over the stages of the differentiation protocol using k=100 as number of clusters. The software is available in the Supplementary Data File 1.

## Data Records

### Data Record 1

The iPSC-related microarray experiments related to this publication have been performed on the Illumina BeadStation 500 platform (Illumina, San Diego, CA, USA) using the Illumina HumanHT-12 BeadChip. The data were uploaded to NCBI GEO and are accessible under (Data Citation 1). The dataset (Data Citation 1) was first released to the public with the publication Matz *et al.*^17^.

### Data Record 2

The hESC-related microarray experiments related to this publication have been performed on the Illumina BeadStation 500 platform (Illumina, San Diego, CA, USA) using the HumanRef-8 Expression BeadChip. The data were uploaded to NCBI GEO and are accessible under (Data Citation 2). The dataset (Data Citation 2) is being released for the first time with the publication of this Data Descriptor.

## Technical Validation

### Transcriptome data

Microarray data were quality controlled via the proprietary Illumina quality control mechanisms. Tables of Pearson correlation coefficients of all samples vs. each other were generated validating the absence of outliers (Tables 2 and 3). Several samples were investigated in triplicates, all others in duplicates. Figure 1b and Figure 1c show that the replicates cluster together as well in the hESC as in the iPSC differentiation experiments as one would expect. Both bootstrap sampling methods implemented in the *pvclust* clustering software confirmed that all clusters within the dendrogram are with one exception (98%) at 100% supported by data. This demonstrates the validity of experiments on the level of whole-genome gene expression.

### k-means clustering to assess differentiation stages and similarity to primary hepatocytes

**Figure 2:**
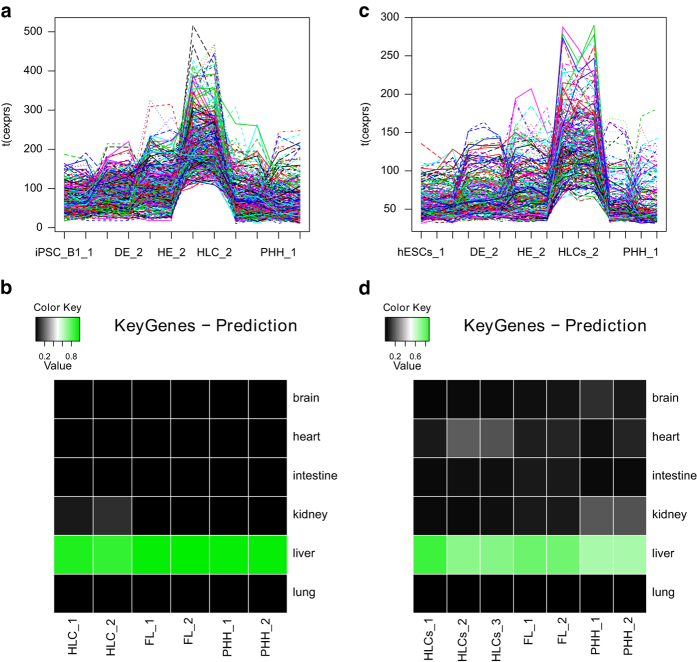
Characterization of hepatocyte-like cells. (**a**) Plot of 407 genes from the k-means cluster#9 over all differentiation stages derived from the iPSCs. The plot demonstrates that this cluster is representative for HLCs. (**b**) KeyGenes tissue classification of for iPSCs k-means Hepatocyte-like-cell (HLC) cluster9 (source: Matz *et al.*^17^). Based on NCBI GEO datasets for human liver, brain, intestine, kidney, lung and heart via the KeyGenes tool a training set for these Illumina microarray platform data was generated. Genes from the HLC cluster#9 resulting from k-means clustering and HLC, fetal liver (FL) and primary human hepatocyte samples were used as test set. (**c**) Plot of 263 genes from the k-means cluster#2 over all differentiation stages derived from the hESCs. The plot demonstrates that this cluster is representative for HLCs. (**d**) KeyGenes tissue-based classification for hESCs k-means Hepatocyte-like-cell (HLC) cluster#2.

Normalized gene expression microarray data of the iPSC differentiation experiments were further investigated via a k-means clustering algorithm. The algorithm split the data into 100 clusters of genes with similar expression over all differentiation stages. Associations of genes with clusters are included in the publication by Matz *et al.*^17^. Several clusters were representative for distinct differentiation stages. Genes from cluster#9 were employed to make a tissue type prediction via the tool KeyGenes^25^ (Figure 2a). Based on the normalized gene expression data of these genes KeyGenes predicted the tissue type “liver” for HLC, fetal liver and PHH samples. Figure 2b demonstrates that genes from cluster#9 have most abundantly peaks at the HLC stages.

**Figure 3:**
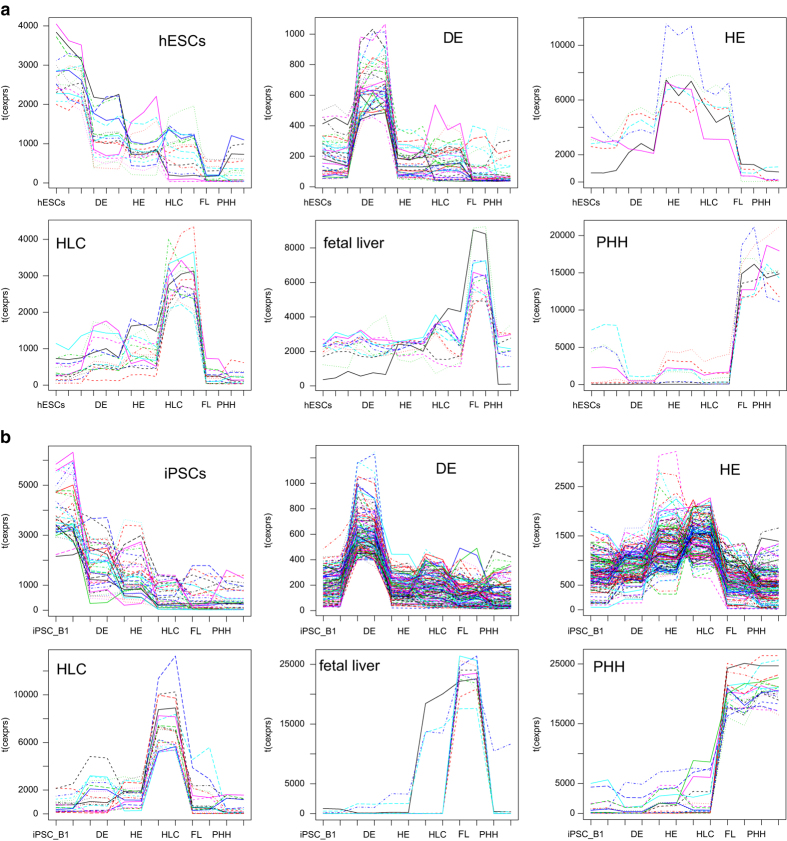
Clusters representative for all stages of differentiation. Via k-means clustering genes were assigned to 100 clusters having similar behaviour during the hepatic differentiation process. The plots in (**a**) show expression of genes in the hESC-differentiation experiments associated with cluster#94 representative for hESCs, cluster#23 containing *SOX17* representative for DE, cluster#64 representative for HE, cluster#77 representative for HLCs, cluster#19 containing AFP representative for fetal liver, cluster#59 containing the liver marker ALB representative for PHHs. The plots in (**b**) show expression of genes in the iPSC-differentiation experiments associated with cluster#68 representative for iPSCs, cluster#81 containing *SOX17* representative for DE, cluster#37 representative for HE, cluster#51 representative for HLCs, cluster#72 containing AFP representative for fetal liver, cluster#91 containing the liver marker *ALB* representative for PHHs.

**Figure 4:**
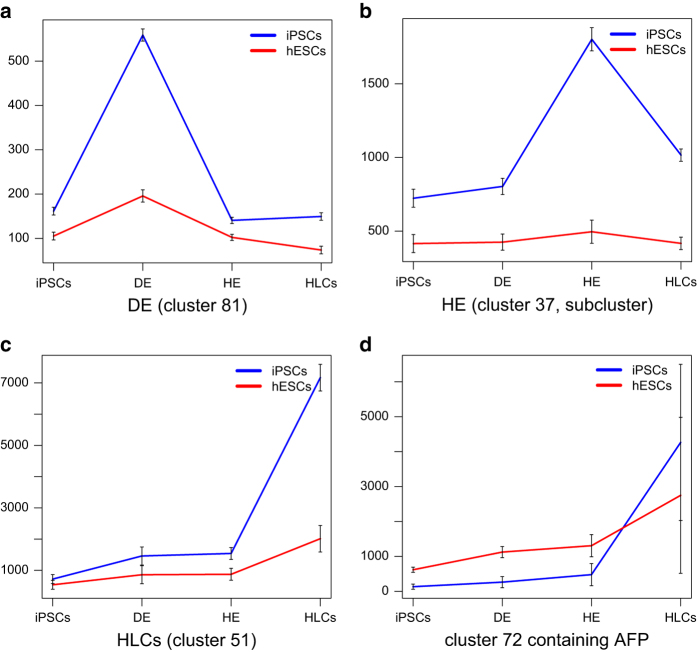
Comparison of clusters representative for DE, HE and HLCs between hESC- and iPSC differentiation. Genes from representative clusters for (**a**) DE (cluster#81), (**b**) HE (cluster#37,subcluster), (**c**) HLCs (cluster#51) and (**d**) cluster #72 containing AFP were compared between hESC- and iPSC-derived lines. Mean and standard error of all genes in the dedicated clusters are shown. The DE, AFP and HLC clusters show maxima at the associated stages in both differentiations. The HE cluster provides a pronounced peak in iPSC-derived cells and a small peak in hESC-derived cells.

Furthermore, k-means clustering provided several stage-specific clusters six of which are shown in Figure 3. These represent stages iPSCs, definite endoderm, hepatic endoderm, HLCs, fetal liver and PHHs and display a gene expression peak at the dedicated stage. They include stage-specific markers which in some cases are already known: *POU5F1/ OCT4* in the iPSC-cluster, *SOX17* in the DE-cluster, *AFP* in the fetal-liver-cluster and *ALB* in the PHH-cluster. In Supplementary Fig. S4F of our previous publication related to the iPSC dataset^17^ of this data descriptor we could confirm PHH-cluster activity of the transcription factors *HNF4α* and *HNF1α* reported by Li *et al.*^15^ as factors inducing hepatocyte differentiation and furthermore reveal the activity of *CTCF, ZFX, FOXA2, FOXA1, CEBPA*. Additionally, these datasets may provide new insights into the differences and similarities of the hepatocyte differentiation processes between hESCs and iPSCs. Figure 4 using marker genes from the representative k-means-clusters shows that the DE stage and HLCs are very similar between hESC- and iPSC-derived differentiations while the HE stage provides a pronounced peak in iPSC-derived cells and a small peak in hESC-derived cells. As a cautionary note, the iPSC and hESC differentiations into HLCs and also the microarray-based transcriptome analyses were not conducted simultaneously, hence the observed minor variations.

## Usage Notes

The microarray experiments related to this publication have been performed on the Illumina BeadStation 500 platform (Illumina, San Diego, CA, USA) but on different BeadChips. The iPSC-derived differentiations were hybridized using the Illumina HumanHT-12 BeadChip while the hESC-derived differentiations were hybridized using the HumanRef-8 Expression BeadChip. The differing chip types should be taken into account when comparing transcriptomics data between hESC-derived and iPSC-derived experiments. Further points which should be considered are: (1) The fetal liver RNA was derived from liver homogenates containing all cells, but the adult liver samples were derived from isolated hepatocytes; (2) the liver disease for which the biopsy was performed could have an influence on the dataset; (3) the two different differentiation protocols used may affect the data.

## Additional information

**How to cite this article:** Wruck, W. & Adjaye, J. Human pluripotent stem cell derived HLC transcriptome data enables molecular dissection of hepatogenesis. *Sci. Data* 5:180035 doi:10.1038/sdata.2018.35 (2018).

**Publisher’s note:** Springer Nature remains neutral with regard to jurisdictional claims in published maps and institutional affiliations.

## Supplementary Material



Supplementary Information

## Figures and Tables

**Table 1:** Samples related to data sets in repositories.

**ID**	**description**	**replicate [#]**	**NCBI GEO sample**	**NCBI GEO accession no.**
hESCs_1	human embryonic stem cells (H1)	1	GSM2683216	GSE100447
hESCs_2	human embryonic stem cells (H1)	2	GSM2683217	GSE100447
hESCs_3	human embryonic stem cells (H1)	3	GSM2683218	GSE100447
DE_hESCs_1	definite endoderm from hESCs	1	GSM2683219	GSE100447
DE_hESCs_2	definite endoderm from hESCs	2	GSM2683220	GSE100447
DE_hESCs_3	definite endoderm from hESCs	3	GSM2683221	GSE100447
HE_hESCs_1	hepatic endoderm from hESCs	1	GSM2683222	GSE100447
HE_hESCs_2	hepatic endoderm from hESCs	2	GSM2683223	GSE100447
HE_hESCs_3	hepatic endoderm from hESCs	3	GSM2683224	GSE100447
HLCs_hESCs_1	hepatocyte-like cells from hESCs	1	GSM2683225	GSE100447
HLCs_hESCs_2	hepatocyte-like cells from hESCs	2	GSM2683226	GSE100447
HLCs_hESCs_3	hepatocyte-like cells from hESCs	3	GSM2683227	GSE100447
Fetal_Liver_1	fetal liver	1	GSM2683228	GSE100447
Fetal_Liver_2	fetal liver	2	GSM2683229	GSE100447
PHH_1	primary human hepatocytes	1	GSM2683230	GSE100447
PHH_2	primary human hepatocytes	2	GSM2683231	GSE100447
iPSCs_1	induced pluripotent stem cells	1	GSM1618658	GSE66282
iPSCs_2	induced pluripotent stem cells	2	GSM1618659	GSE66282
DE_iPSCs_1	definite endoderm from iPSCs	1	GSM1618660	GSE66282
DE_iPSCs_2	definite endoderm from iPSCs	2	GSM1618661	GSE66282
HE_iPSCs_1	hepatic endoderm from iPSCs	1	GSM1618662	GSE66282
HE_iPSCs_2	hepatic endoderm from iPSCs	2	GSM1618663	GSE66282
HLCs_iPSCs_1	hepatocyte-like cells from iPSCs	1	GSM1618664	GSE66282
HLCs_iPSCs_2	hepatocyte-like cells from iPSCs	2	GSM1618665	GSE66282
fetal_liver_1	fetal liver	1	GSM1618666	GSE66282
fetal_liver_2	fetal liver	2	GSM1618667	GSE66282
PHH_1	primary human hepatocytes	1	GSM1618668	GSE66282
PHH_2	primary human hepatocytes	2	GSM1618669	GSE66282

**Table 2:** Pearson correlation coefficients of hESC-derived transcriptome data of all samples vs. each other.

sample	hESCs_1	hESCs_2	hESCs_3	DE_1	DE_2	DE_3	HE_1	HE_2	HE_3	HLCs_1	HLCs_2	HLCs_3	Fetal_Liver_1	Fetal_Liver_2	PHH_1	PHH_2
hESCs_1	1.0000	0.9927	0.9913	0.9489	0.9455	0.9463	0.9534	0.9539	0.9564	0.9117	0.9057	0.9081	0.6798	0.6787	0.6062	0.6079
hESCs_2	0.9927	1.0000	0.9964	0.9483	0.9458	0.9463	0.9530	0.9538	0.9567	0.9094	0.9081	0.9100	0.6910	0.6919	0.6103	0.6113
hESCs_3	0.9913	0.9964	1.0000	0.9477	0.9463	0.9460	0.9539	0.9544	0.9563	0.9177	0.9182	0.9206	0.6903	0.6907	0.6114	0.6124
DE_1	0.9489	0.9483	0.9477	1.0000	0.9963	0.9968	0.9631	0.9631	0.9640	0.9308	0.9270	0.9273	0.6743	0.6725	0.6133	0.6149
DE_2	0.9455	0.9458	0.9463	0.9963	1.0000	0.9978	0.9620	0.9613	0.9618	0.9255	0.9255	0.9281	0.6663	0.6645	0.6097	0.6115
DE_3	0.9463	0.9463	0.9460	0.9968	0.9978	1.0000	0.9597	0.9586	0.9601	0.9213	0.9200	0.9220	0.6662	0.6643	0.6113	0.6135
HE_1	0.9534	0.9530	0.9539	0.9631	0.9620	0.9597	1.0000	0.9981	0.9974	0.9453	0.9422	0.9447	0.6805	0.6799	0.6224	0.6234
HE_2	0.9539	0.9538	0.9544	0.9631	0.9613	0.9586	0.9981	1.0000	0.9975	0.9458	0.9432	0.9451	0.6831	0.6825	0.6211	0.6223
HE_3	0.9564	0.9567	0.9563	0.9640	0.9618	0.9601	0.9974	0.9975	1.0000	0.9411	0.9379	0.9398	0.6812	0.6809	0.6211	0.6226
HLCs_1	0.9117	0.9094	0.9177	0.9308	0.9255	0.9213	0.9453	0.9458	0.9411	1.0000	0.9871	0.9834	0.6912	0.6889	0.6063	0.6086
HLCs_2	0.9057	0.9081	0.9182	0.9270	0.9255	0.9200	0.9422	0.9432	0.9379	0.9871	1.0000	0.9972	0.6931	0.6923	0.6121	0.6149
HLCs_3	0.9081	0.9100	0.9206	0.9273	0.9281	0.9220	0.9447	0.9451	0.9398	0.9834	0.9972	1.0000	0.6854	0.6849	0.6110	0.6139
Fetal_Liver_1	0.6798	0.6910	0.6903	0.6743	0.6663	0.6662	0.6805	0.6831	0.6812	0.6912	0.6931	0.6854	1.0000	0.9979	0.7384	0.7364
Fetal_Liver_2	0.6787	0.6919	0.6907	0.6725	0.6645	0.6643	0.6799	0.6825	0.6809	0.6889	0.6923	0.6849	0.9979	1.0000	0.7422	0.7403
PHH_1	0.6062	0.6103	0.6114	0.6133	0.6097	0.6113	0.6224	0.6211	0.6211	0.6063	0.6121	0.6110	0.7384	0.7422	1.0000	0.9973
PHH_2	0.6079	0.6113	0.6124	0.6149	0.6115	0.6135	0.6234	0.6223	0.6226	0.6086	0.6149	0.6139	0.7364	0.7403	0.9973	1.0000

**Table 3:** Pearson correlation coefficients of iPSC-derived transcriptome data of all samples vs. each other.

sample	iPSC_B1_1	iPSC_B1_2	DE_1	DE_2	HE_1	HE_2	HLC_1	HLC_2	fetal_liver_1	fetal_liver_2	PHH_1	PHH_2
iPSC_B1_1	1.0000	0.9948	0.9356	0.9387	0.9502	0.9490	0.9155	0.9214	0.8150	0.8244	0.7413	0.7336
iPSC_B1_2	0.9948	1.0000	0.9419	0.9448	0.9541	0.9527	0.9228	0.9274	0.8236	0.8331	0.7512	0.7431
DE_1	0.9356	0.9419	1.0000	0.9980	0.9202	0.9124	0.9546	0.9496	0.8191	0.8285	0.7444	0.7363
DE_2	0.9387	0.9448	0.9980	1.0000	0.9238	0.9165	0.9548	0.9508	0.8199	0.8297	0.7453	0.7371
HE_1	0.9502	0.9541	0.9202	0.9238	1.0000	0.9966	0.9225	0.9363	0.8360	0.8379	0.7643	0.7581
HE_2	0.9490	0.9527	0.9124	0.9165	0.9966	1.0000	0.9165	0.9311	0.8328	0.8345	0.7639	0.7582
HLC_1	0.9155	0.9228	0.9546	0.9548	0.9225	0.9165	1.0000	0.9956	0.8399	0.8488	0.7714	0.7642
HLC_2	0.9214	0.9274	0.9496	0.9508	0.9363	0.9311	0.9956	1.0000	0.8440	0.8510	0.7753	0.7685
fetal_liver_1	0.8150	0.8236	0.8191	0.8199	0.8360	0.8328	0.8399	0.8440	1.0000	0.9941	0.8684	0.8624
fetal_liver_2	0.8244	0.8331	0.8285	0.8297	0.8379	0.8345	0.8488	0.8510	0.9941	1.0000	0.8662	0.8593
PHH_1	0.7413	0.7512	0.7444	0.7453	0.7643	0.7639	0.7714	0.7753	0.8684	0.8662	1.0000	0.9974
PHH_2	0.7336	0.7431	0.7363	0.7371	0.7581	0.7582	0.7642	0.7685	0.8624	0.8593	0.9974	1.0000
