# Supplementary material for: Human pluripotent stem cell derived HLC transcriptome data enables molecular dissection of hepatogenesis
Source: Sci Data. 2018 Mar 13;5:180035. doi: 10.1038/sdata.2018.35 (PMC5848791; doi:10.1038/sdata.2018.35)
Supplement: Supplementary Information [file sdata201835-s2.zip › wruck_etal_analysis_software/Description_ software_analysis_HLCs.doc]

# Description of R software for analysis of Illumina Beadchip data of hepatocyte-like cells (HLCs) related to Wruck *et al.* “Human pluripotent stem cell derived HLC transcriptome data enables molecular dissection of hepatogenesis”

## Software for analysis of iPSC-derived and hESC-derived HLCs

R source code: ***lumi_evalGEO_hesc_ipsc_hlcs.R***

***Required R packages: lumi, affy, pvclust***

The software reads the non-normalized data files which can be downloaded from NCBI GEO (accession nos. GSE66282 and GSE100447) and unzipped (*GSE66282_non_normalized.txt,* for hESCs: *GSE100447_non-normalized.txt*). Sample names and associations with array sections are read from the sample lookup file (*samples_ipsc_hlc.txt*, for hESCs: *samples_hesc_hlc.txt*). Background correction and normalization are performed via the *lumi* R package. Normalized data in the GEO format containing expression values and detection p values is written to file *GEOnormalizedData_ipsc_hlc.txt*, for hESCs: *GEOnormalizedData_hesc_hlc.txt*. The analyses in the manuscript were based upon the Illumina gene profiles which also contained bead standard error data. With the standard error data background correction could be performed via the *lumiB* method which is not possible with the GEO data. The GEO data does not have a slot for the bead standard error data. Therefore the background was adjusted with a slightly different but comparable method *bg.adjust* from the *affy* package. However, except some marginal differences the results are the same.

Bootstrap clustering via pvclust

Via the R package *pvclust* a bootstrap clustering is performed. Parameter nboot=1000 will run approximately 1 hour on an average PC from 2013, for testing purposes nboot=100 may be used and will finish considerably faster.

k-means clustering

k-means clustering is initialized with a random number and therefore is not directly reproducible.

Therefore we have saved the generated clusters used for the publication in file *clusters_kmeans_ipsc.txt* (for hESCs: *clusters_kmeans_hesc.txt*) and read them for reproducibility. To generate a new k-means clustering set KMEANSFROMFILE to 0.

Comparison of DE-,HE-, AFP- and HLC-specific clusters between hESC- and iPSC-based differentiations

The stages are compared based on the ipsc-based k-means clustering using clusters DE (81), HE (37), containing AFP (72) and HLC (51). Cluster 37 was very big containing also genes with peaks in the HLC stage and was therefore reduced to marker genes which were in average more than 1.33-fold higher in HE than in HLC. For iPSC- and hESC-derived replicates the mean was taken and afterwards mean, standard deviation of all genes associated with a cluster was calculated and plotted. Error bars display the standard errors.

>sessionInfo()

R version 3.2.2 (2015-08-14)

Platform: x86_64-w64-mingw32/x64 (64-bit)

Running under: Windows 7 x64 (build 7601) Service Pack 1

locale:

[1] LC_COLLATE=English_United States.1252

[2] LC_CTYPE=English_United States.1252

[3] LC_MONETARY=English_United States.1252

[4] LC_NUMERIC=C

[5] LC_TIME=English_United States.1252

attached base packages:

[1] parallel stats	graphics grDevices utils	datasets methods

[8] base

other attached packages:

[1] affy_1.48.0	pvclust_2.0-0	lumi_2.22.1

[4] Biobase_2.30.0	BiocGenerics_0.16.0
